# Antiplasmodial profile of selected compounds from Malaria Box: in vitro evaluation, speed of action and drug combination studies

**DOI:** 10.1186/s12936-019-3069-3

**Published:** 2019-12-30

**Authors:** Guilherme Eduardo de Souza, Renata Vieira Bueno, Juliana Oliveira de Souza, Camila Lima Zanini, Fábio Cardoso Cruz, Glaucius Oliva, Rafael Victório Carvalho Guido, Anna Caroline Campos Aguiar

**Affiliations:** 1 Sao Carlos Institute of Physics, University of Sao Paulo, Av. Joao Dagnone, 1100 Jardim Santa Angelina, São Carlos, SP 13563-120 Brazil; 2 Department of Pharmacology, Federal University of São Paulo, Rua Botucatu 862, São Paulo, SP 04023-062 Brazil

**Keywords:** Antimalarial, Drug resistance, Combination therapy, Drug development, Malaria Box

## Abstract

**Background:**

Artemisinin-based combination therapy (ACT) is used as the first-line treatment of uncomplicated malaria caused by the *Plasmodium falciparum* parasite and chloroquine-resistant *Plasmodium vivax* parasites. Evidence of resistance to ACT has been reported in Cambodia, and without new and effective anti-malarial agents, malaria burden and mortality will rise.

**Methods:**

The used MolPrint 2D fingerprints and the Tanimoto similarity index were used to perform a structural similarity search within the Malaria Box collection to select diverse molecular scaffolds that are different from artesunate. Next, the inhibitory potency against the *P. falciparum* 3D7 strain (SYBR Green I inhibition assay) and the cytotoxicity against HepG2 cells (MTT and neutral red assays) were evaluated. Then, the speed of action, the combination profile of selected inhibitors with artesunate, and the *P. berghei* in vivo activity of the best compounds were assessed.

**Results:**

A set of 11 structurally diverse compounds from the Malaria Box with a similarity threshold of less than 0.05 was selected and compared with artesunate. The in vitro inhibitory activity of each compound confirmed the reported potencies (IC_50_ values ranging from 0.005 to 1 µM). The cytotoxicity of each selected compound was evaluated and used to calculate the selectivity index (SI values ranging from 15.1 to 6100). Next, both the speed of action and the combination profile of each compound with artesunate was assessed. Acridine, thiazolopyrimidine, quinoxaline, benzimidazole, thiophene, benzodiazepine, isoxazole and pyrimidoindole derivatives showed fast in vitro inhibitory activity of parasite growth, whereas hydrazinobenzimidazole, indenopyridazinone and naphthalenone derivatives were slow-acting in vitro inhibitors. Combinatory profile evaluation indicated that thiazolopyrimidinone and benzodiazepine derivatives have an additive profile, suggesting that the combination of these inhibitors with artesunate is favourable for in vitro inhibitory activity. The remaining compounds showed an antagonistic combinatory profile with artesunate. The collected data indicated that the indenopyridazinone derivative, a bc_1_ complex inhibitor, had a similar association profile in combination with proguanil when compared to atovaquone combined with proguanil, thereby corroborating the correlation between the molecular target and the combination profile. Lastly, the in vivo activity of the thiazolopyrimidinone and benzodiazepine derivatives were assessed. Both compounds showed oral efficacy at 50 mg/kg in a mouse model of *Plasmodium berghei* malaria (64% and 40% reduction in parasitaemia on day 5 post-infection, respectively).

**Conclusions:**

The findings in this paper shed light on the relationship among the speed of action, molecular target and combinatory profile and identified new hits with in vivo activity as candidates for anti-malarial combination therapy.

## Background

Malaria is a tropical disease with the highest mortality rate in low-income countries. In 2017, 212 million new cases and 435,000 deaths were reported [1]. *Plasmodium falciparum* is responsible for the majority of malaria deaths globally and is the most prevalent species in sub-Saharan Africa [2].

Artemisinin-based combination therapy (ACT) is the first-line treatment for uncomplicated malaria caused by the *P. falciparum* parasite and chloroquine-resistant *Plasmodium vivax* parasites [3]. The combination of an artemisinin derivative with active compounds with different mechanisms of action improves the efficacy of the artemisinin analogue, reduces the treatment course, and decreases the resistance development potential [4]. However, since 2008, evidence of resistance to ACT has been reported in Cambodia, and patients with delayed parasite clearance under artemisinin treatment have been routinely identified [5–10]. Epidemiologic studies in southeast Asia and along India’s borders have been monitoring the parasitic strains that are resistant to both artemisinin derivatives and partner drugs, such as mefloquine and piperaquine [11, 12]. The spread of drug-resistant parasites can lead to an overall reduction in drug efficacy and treatment failure [7]. In the worst-case scenario, this spread could lead to an epidemic of drug-resistant malaria. Hence, malaria burden and mortality may significantly increase in the absence of new and effective drugs.

Medicines for Malaria Venture (MMV) is a leading product development partnership in the field of anti-malarial drug research and development. Aiming to catalyse a robust drug discovery process, MMV released in 2013 the Malaria Box, a collection of 400 free access compounds that show inhibitory activity against *P. falciparum* blood stages [13]. In this work, the antiplasmodial profile of 11 chemically diverse molecules selected from the Malaria Box was investigated. The compounds had their inhibitory activity against *P. falciparum* confirmed, their cytotoxicity evaluated and their speed-of-action and combination profile with artesunate assessed. Lastly, two promising compounds were selected for in vivo evaluation to complete the activity profile.

## Methods

### Compound selection

The 2D structures of artesunate and the 400 compounds from the Malaria Box were retrieved from the ChEMBL database (https://www.ebi.ac.uk/chembl), and their MolPrint 2D binary fingerprints were generated using the chemoinformatics software Canvas (Schrödinger, LLC, New York, NY) [14, 15]. The binary fingerprints of the 401 compounds were used to run a hierarchical clustering analysis using the Tanimoto similarity index and the average linkage method to calculate the distance between all inter-cluster pairs [14]. Eleven molecules from the Malaria Box, representing eight different clusters, were selected based on chemical diversity, physicochemical properties, and commercial availability from the same chemical supplier for inhibitory activity evaluation. In addition, the Molprint 2D fingerprints and Tanimoto similarity indices were used to compare artesunate and the selected compounds through a similarity matrix, with similarity indices varying from 0 to 1 [14]. The 11 molecules were purchased from the Ambinter, and stock solutions at a concentration of 20 mM were prepared in 100% DMSO before the in vitro assays.

### Maintenance of in vitro culture

The *P. falciparum* 3D7 strain was kept in culture in a humidified incubator at 37 °C in RPMI-1640 medium with 25 mM NaHCO_3_, 25 mM HEPES (pH 7.4), 11 mM d-glucose, 3.67 mM hypoxanthine and 25 µg/mL gentamicin, supplemented with 0.5% (m/v) AlbuMAX II. The culture medium was changed daily, and the parasitaemia was maintained below 10% with 2.5% haematocrit in human erythrocytes [16].

### SYBR Green I inhibition assay for the asexual stages of *P. falciparum*

The parasites were synchronized through sterile 5% (m/v) d-sorbitol treatment over 10 min at 37 °C for the enrichment of ring-stage parasites [17]. The cultures were pelleted by centrifugation at 600×*g* over 5 min. The parasitaemia was determined by microscope analysis of thin blood smears stained with Giemsa 10% (v/v) after methanol fixation. The initial parasitaemia was calculated from 1000 red blood cells (RBCs), and cultures were diluted to 0.5% parasitaemia and 2% haematocrit by adding the appropriate volumes of erythrocytes and medium. Parasite aliquots of 180 µL were distributed into 96-well plates previously prepared with 20 µL aliquots of a tenfold concentrated compound. Negative and positive control wells corresponding to non-parasitized erythrocytes and parasite cultures in the absence of compounds were set in parallel. The DMSO concentration was maintained below 0.05% (v/v). The plates were incubated for 72 h at 37 °C in a humidified incubator with a gas mixture of 90% N_2_, 5% O_2_ and 5% CO_2_. After incubation, the culture medium was removed, and the cells were resuspended in 100 µL PBS buffer (116 mM NaCl, 10 mM Na_2_HPO_4_, 3 mM KH_2_PO_4_) and lysed with 100 µL lysis buffer (20 mM Tris base, 5 mM EDTA, 0.0008% (v/v) Triton X-100, 0.008% (m/v) saponin, pH 8.0) containing 0.002% (v/v) SYBR Green I. Plates were incubated at room temperature for 30 min, and the fluorescence corresponding to the parasitic density was determined using a SpectraMAX Gemini EM plate reader (Molecular Devices Corp., Sunnyvale, CA) (excitation at 485 nm, emission at 535 nm) [18]. The half maximal inhibitory concentration (IC_50_^Pf^) was determined by non-linear regression analysis of the resulting concentration-response curve using the software Origin 2016 (OriginLab Corporation).

### Speed of action studies

The speed of action of the anti-malarial candidates was verified by the incubation of the synchronized parasites at the ring stage in the presence of compounds at a concentration that was tenfold greater than the IC_50_ value. Parasites in thin blood smears were observed by microscopy after 0, 8, 16, 24 and 36 h of incubation [19].

### Prediction of drug absorption

A multivariate approach developed by Egan et al. [20] was used to determine whether the investigated compounds could have membrane permeability issues. Calculated values of log P [21] and polar surface area (PSA) were used as independent parameters underlying the lipid-based diffusion to define value ranges that correlated with good absorption [20]. log P and PSA were plotted for each compound, and a 99% confidence ellipse outlined the area for well-absorbed compounds.

### Combination assays with artesunate

Combinations of anti-malarial candidates with artesunate, an artemisinin derivative, were tested in fixed molar ratios based on the individual IC_50_ values that were previously determined by the SYBR Green I assay. Traditionally, a line (isobole) of additivity is used to distinguish between additive, synergic, and antagonistic interaction profiles. However, this additive profile is valid only for the interaction of compounds with a constant ratio of potencies, which is not the case for the anti-malarial compounds studied in this work (Additional file 1: Fig. S3). Thus, a systematic consideration of additivity ranges was included in the isobologram analysis, as previously described by Grabovsky and Tallarida [22]. Concentration-response curves of individual compounds were used in the calculation of each pair’s additivity range (area within the upper and lower limits in Figs. 4 and 5). Fractional inhibitory concentration (FIC_50_) pairs plotted as points inside the additivity range indicate the expected sum of each compound effect, while points above and below the range indicate antagonistic and synergic interactions, respectively. The selected inhibitors were combined with artesunate in the IC_50_ equivalent ratios of 1:4, 2:3, 1:1, 3:2 and 4:1 for the construction of the isobolograms [23].

### Hepatocarcinoma cell cultures and cytotoxicity evaluation

Hepatocarcinoma cells (HepG2) were cultivated in RPMI medium supplemented with 10% (v/v) fetal bovine serum and 0.2% (v/v) penicillin/streptomycin. The antibiotics were added to the medium to eliminate the potential interference of microbial contamination. Cells were cultivated at 37 °C and 5% CO_2_; the supplemented medium was changed every 2 days.

For the experimental procedures, cells were trypsinized and transferred to a 96-well plate at 30,000 cells per well and incubated at 37 °C overnight for cell adhesion. Then, serial dilutions of the inhibitor candidates were added to the plate. Cells without any compounds were used as positive growth controls. The plate was incubated at 37 °C and 5% CO_2_ for 72 h. After incubation, the highest compound concentration to be considered (highest concentration without precipitation) was observed by microscopy. Cytotoxicity was evaluated by two different methods. The first is a colorimetric assay based on metabolic cell activity in the presence of 3-(4,5-dimethylthiazol-2-yl)-2,5-diphenyltetrazolium bromide (MTT) [24]. Briefly, mitochondrial enzymes can convert the MTT dye into the purple insoluble compound formazan. To each well, 20 µL MTT at 5 mg/mL was added, followed by incubation at 37 °C for 3 to 5 h. After incubation, the supernatant was removed, and formazan crystals were solubilized in 100 µL DMSO. The absorbance, which is proportional to the number of viable cells, was determined using a SpectraMAX Plus 384 plate reader (Molecular Devices Corp., Sunnyvale, CA) (λ = 570 nm) [25]. The second method is based on the uptake of neutral red, a dye that is retained inside the lysosomes of viable cells. To each well, after removal of culture media, 200 µL neutral red medium 40 µg/mL was added, followed by incubation at 37 °C for 3 to 5 h. After incubation, supernatant was dispensed, and 200 µL 0.5% (v/v) formaldehyde in 1% (m/v) CaCl_2_ was added to remove excess of the dye for 5 min. Supernatant was dispensed, and 100 µL alcohol-acid solution (50% (v/v) ethanol, 1% (v/v) glacial acetic acid) was added, and the plate was stirred until a homogeneous solution was formed. The absorbance was determined using a SpectraMAX Plus 384 plate reader (Molecular Devices Corp., Sunnyvale, CA) (λ = 540 nm) [26]. For both methods, the half maximal inhibitory concentration (IC_50_^HepG2^) was determined by non-linear regression analysis of the resulting concentration-response curve using the software Origin 2016 (OriginLab Corporation).

### In vivo assay against *Plasmodium berghei*

A suppressive parasite growth test was performed in mice infected with *P. berghei* NK65 strain (originally received from the New York University Medical School), as described previously [27], with some modifications. Briefly, adult Swiss outbred mice (20 ± 2 g weight) were intraperitoneally inoculated with 1 × 10^5^ red blood cells infected with *P. berghei*. The infected mice were maintained together for at least 2 h and then randomized into groups of 5 animals per cage, which were subsequently administered 50 mg/kg of each compound diluted in 3% (v/v) DMSO by oral gavage daily for 3 days. Two control groups were used in parallel: one was treated with CQ (20 mg/kg) and the other was treated with the vehicle. Blood smears from mouse tails were prepared on days 5 and 7 post-infection and then fixed with methanol, stained with Giemsa 10% (v/v), and examined under the microscope. Parasitaemia was evaluated and the percent inhibition of parasite growth was calculated in relation to the untreated group (considered 100% growth) using the following equation:

$$ [(\text{C} - \text{T}/\text{C})] \times 100, $$

where C is the parasitemia in the control group and T is the parasitaemia in the treated group. The use of laboratory animals was approved by the Ethics Committee for Animal Use of Universidade Federal do Estado de São Paulo, UNIFESP (CEUA N 6630080816).

## Results

### Malaria Box compound selection

The 400 compounds that comprise the Malaria Box were selected considering the chemical diversity, potency, and commercial availability of an initial set of compounds containing 19,873 unique hits [13]. Based on that, chemoinformatic approaches were used to select representative compounds from the Malaria Box that were as dissimilar as possible to artesunate. In order to do this, the Molprint 2D fingerprints and Tanimoto similarity indices were applied to construct a similarity matrix and compare artesunate with the Malaria Box compounds. The 400 compounds from the Malaria Box were grouped into 40 clusters based on their atomic connectivity and topological distances. The similarity indices varied from 0 (dissimilar scaffold) to 1 (identical scaffold). A similarity threshold of less than 0.05 was employed and 11 representative scaffolds were selected based on chemical diversity and commercial availability from the same supplier for inhibitory activity evaluation.

**Fig. 1:**
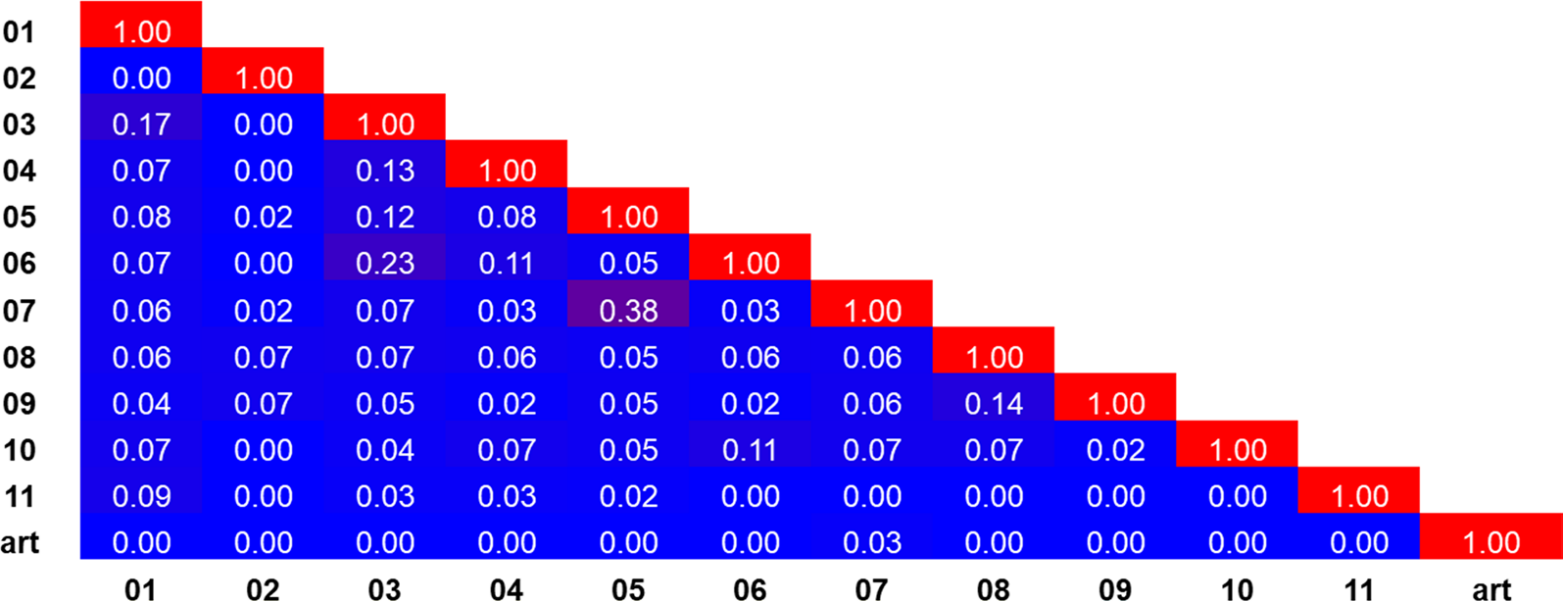
Similarity matrix for the eleven compounds (**1**–**11**) selected from the Malaria Box and artesunate (art). The similarity indices vary from 0 (minimum) to 1 (maximum) and are coloured as shades of blue and red, respectively

This set of 11 compounds contains compounds that belong to eight different clusters with diverse physicochemical properties (Additional file 1: Figs S1 and S2). The selected scaffolds are very diverse and include diazines, piperidines, piperazines, benzimidazoles, thiophene heterocycles, halogen substituted phenyl rings, and aliphatic functional groups, such as carboxamides and carboxylates (Table 1). The similarity matrix of the 11 representative compounds and artesunate according to the Tanimoto metric and MolPrint2D fingerprint is represented in Fig. 1. According to the similarity matrix, the selected compounds share a structural similarity of less than 3% with artesunate. Among the selected dataset, compounds **5** (**MMV020439**) and **7** (**MMV007574**) are the most similar (38% similarity). Both compounds were grouped in cluster 23 because they share a thiophene carboxamide substituent (Additional file 1: Figs S1 and S2), whereas compounds **3** (**MMV007224**) and **6** (**MMV665934**) share 23% similarity due to the presence of a 4-bromophenyl substituent. The similarity index amongst the remaining set of compounds is less than 20%.

**Table 1:** Inhibitory activity against *P. falciparum* (reported and evaluated IC_50_^Pf^, 3D7 strain), hepatocarcinoma cells using MTT assay

#	Code	Structure	Reported IC_50_^Pf^ (µM)	IC_50_^Pf^ (µM) Average (confidence interval-95%)	IC_50_^HepG2, MTT^ (µM) Average (confidence interval-95%)	IC_50_^HepG2, NR^ (µM) Average (confidence interval-95%)	SI^MTT^ Average (confidence interval- 95%)	SI^NR^ Average (confidence interval-95%)
01	**MMV006172**		0.142	0.074 (0.072–0.076)	3.21 (3.18–3.24)	3.34 (3.28–3.40)	43.4 (42.6–44.2)	45.1 (44.6–45.6)
02	**MMV665971**		0.489	0.40 (0.39–0.41)	22 (16–28)	22 (18–26)	60 (50–70)	55 (46–64)
03	**MMV007224**		1.06	0.34 (0.29–0.39)	5.1 (4.5–5.7)	> 6.25	15.1 (14.6–15.6)	> 16
04	**MMV666607**		0.142	0.44 (0.36–0.52)	> 6.25	> 6.25	> 12	> 12
05	**MMV020439**		0.512	0.25 (0.16–0.34)	90 (80–100)	> 100	340 (280–400)	> 290
06	**MMV665934**		1.07	1.2 (0.5–1.9)	> 12.5	> 12.5	> 6.6	> 6.6
07	**MMV007574**		0.826	1.0 (0.8–1.2)	> 25	> 25	> 21	> 21
08	**MMV085203**		0.0053	0.0055 (0.005–0.006)	> 50	33 (28–38)	> 8300	6100 (5800–6400)
09	**MMV085583**		0.237	0.17 (0.14–0.20)	> 12.5	> 12.5	> 63	> 63
10	**MMV018984**		0.693	0.85 (0.78–0.92)	nd	nd	nd	nd
11	**MMV019871**		0.345	1.6 (1.5–1.7)	> 50	> 50	> 29	> 29
Artesunate			0.008	0.017 (0.016–0.018)	110 (80–140)	110 (80–140)	7000 (6000–8000)	7000 (6000–8000)
2 + artesunate			–	0.015 (0.014–0.016)	15.70 (15.64–15.74)	13.0 (12.3–13.7)	1030 (940–1120)	850 (820–880)
9 + artesunate			–	0.013 (0.011–0.015)	> 12.5	> 12.5	> 840	> 840

(IC_50_^HepG2, MTT^) and neutral red assay (IC_50_^HepG2, NR^), and selectivity index (SI^MTT^ and SI^NR^) values of the selected compounds from the Malaria Box. Inhibitory activity, cytotoxicity and selectivity were also evaluated for artesunate and the pairs **2** + artesunate and **9** + artesunate, which showed additive combination profile

Nd, not determined; the compound was not soluble in the assay conditions

### Inhibitory activity and cytotoxicity evaluation of selected Malaria Box compounds

The 11 selected compounds were purchased from Ambinter, a chemical compound supplier and confirmed the reported in vitro inhibitory activity against the intra-erythrocytic form of *P. falciparum* (3D7 strain) (Table 1 and Additional file 1: Fig. S3). In general, there was a good agreement between the reported and evaluated potency values (*r*^2^ = 0.8) (Table 1, Additional file 1: Fig. S4). The measured IC_50_ values ranged from 0.0056 µM (pIC_50_ = 8.3) to 1.6 µM (pIC_50_ = 5.8) and were distributed across the range of two orders of magnitude (Table 1 and Additional file 1: Fig. S4). Therefore, the set of selected scaffolds includes representative bioactive compounds of the Malaria Box. Compound **8,** a naphthalen-1(2H)-one derivative, was the most potent inhibitor among the evaluated compounds (IC_50_^Pf^ = 0.0056 µM), whereas compound **11**, a pyrimido[5,4-b]indol-4-amine derivative, was the less potent inhibitor (IC_50_^Pf^ = 1.6 µM).

The cytotoxicity of the selected compounds was determined using a human hepatic cell line (HepG2) with the MTT assay [24] and the neutral red assay [26]. The selectivity index (SI) was assessed as the ratio between the inhibitory activity against the hepatocytes (IC_50_^HepG2^) and *P. falciparum* (IC_50_^Pf^). Values of SI > 10 indicate a favourable safety window between the effective concentration against the parasite and the toxic concentration to the human cell. Data from MTT and neutral red assays showed no significative discrepancies. The compounds were moderately to highly selective, with SI values ranging from 15.1 to 6100 (Table 1). The IC_50_^HepG2^ for compound **10** was not determined due to the low solubility in the assay conditions.

### Speed of action analysis

**Fig. 2:**
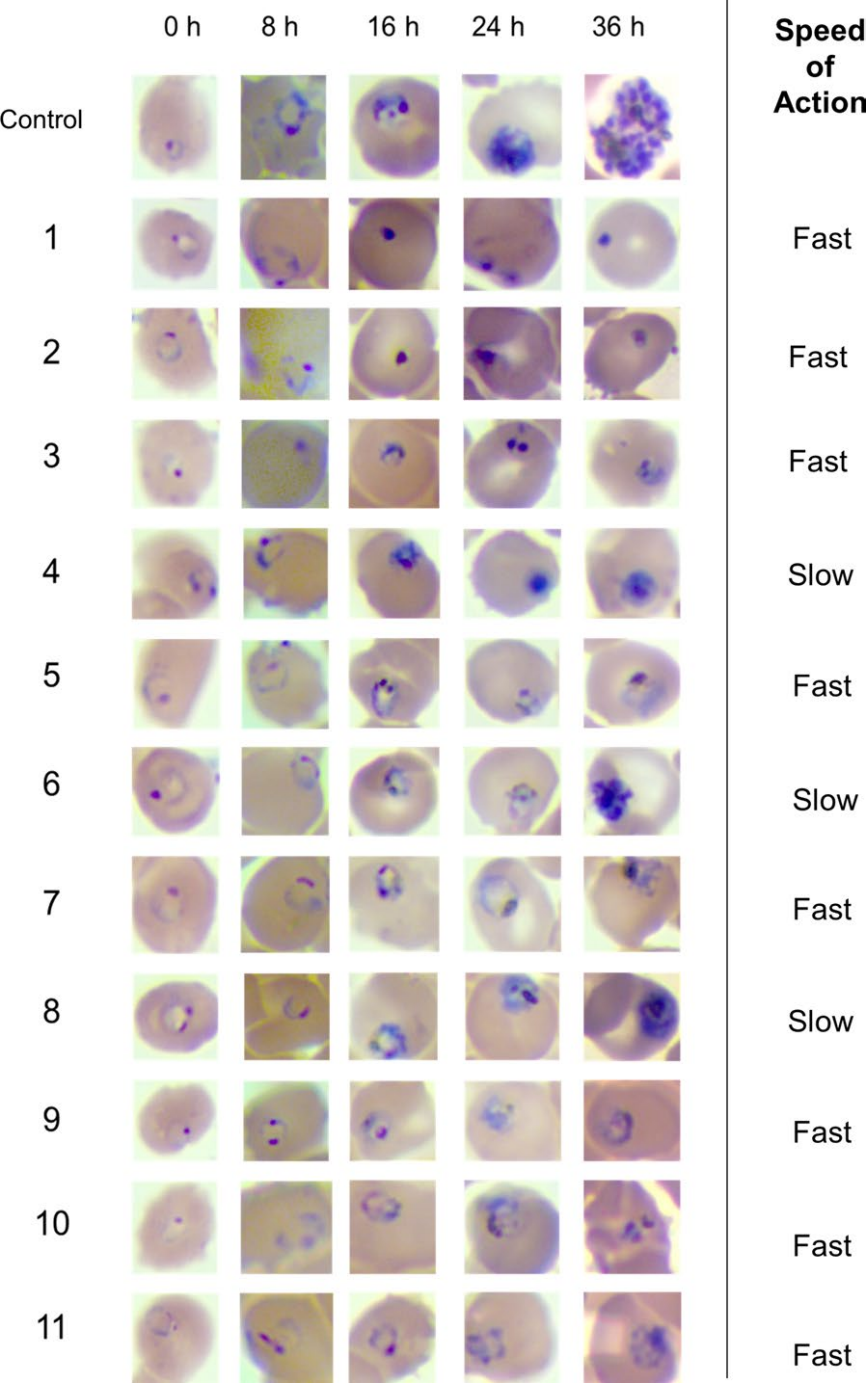
Speed of action investigation for compounds **1**–**11** selected from the Malaria Box. One maturation cycle was evaluated for the control condition (absence of inhibitors), ranging from ring-stage (0 h) to trophozoite (24 h) to schizont (36 h). Compounds were considered fast inhibitors if morphology development was interrupted at early stages, and slow inhibitors if morphology development reached late stages

To further investigate the antiplasmodial profile of the selected inhibitors, the speed of action was assessed against the asexual intraerythrocytic stages of *P. falciparum*. Over the incubation time of 36 h, one complete maturation cycle of the parasite was observed (from ring to schizont stage) under the assay conditions (control row, Fig. 2). Compounds **1** and **2** induced pyknotic nuclei formation, the irreversible chromatin condensation of a necrotic or apoptotic cell, after 16 h of incubation. For compounds **3**, **5**, **7**, **9**, **10** and **11**, the microscopic analysis of the parasite morphology indicated that the parasites exhibited punctual shapes after 16 h, suggesting that the parasite development stalled in the ring stage up to 36 h of incubation. However, in the presence of **4**, **6**, and **8**, young trophozoites were observed after 36 h of incubation. Thus, the findings indicate that compounds **1**–**3**, **5**, **7**, **9**–**11** are fast-acting inhibitors, whereas compounds **4**, **6**, and **8** are slow-acting inhibitors of in vitro parasite growth.

**Fig. 3:**
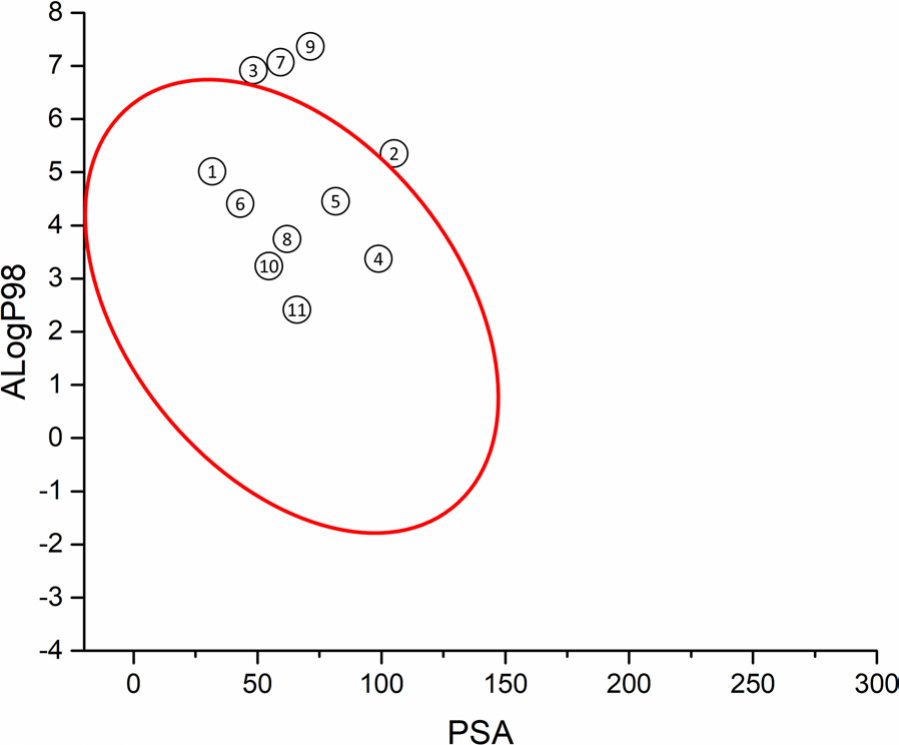
Plot of PSA vs. AlogP98 for anti-malarial compounds, calculated using BIOVIA Draw 18.1. The red ellipse represents the 99% confidence limit for drugs with good absorption [20]

Using drug absorption prediction analysis, seven compounds (**1**, **4**–**6**, **8**, **10**, and **11**) fell into the 99% confidence ellipse for well-absorbed drugs (Fig. 3). Accordingly, the computational analysis suggested that these inhibitors have favourable permeability properties, thereby suggesting that permeability may not be an issue related to the observed speed of action for this set of inhibitors.

### Isobologram analysis

**Fig. 4:**
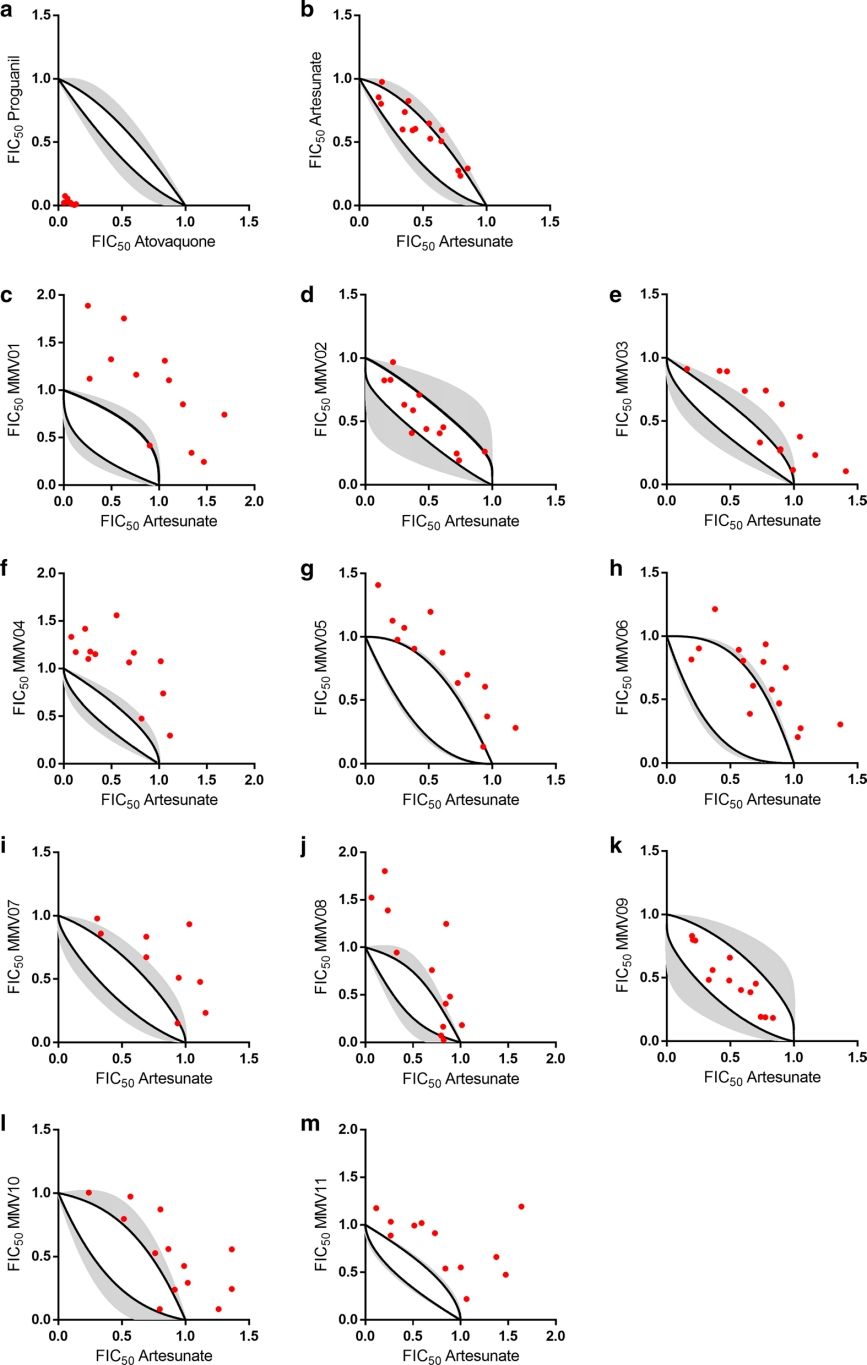
Isobolograms of controls and selected Malaria Box compounds. **a** Atovaquone with proguanil and **b** artesunate with itself were used as controls for synergic and additive profiles, respectively. **c**–**m** Isobolograms of the 11 selected compounds in combination with artesunate. Black lines and grey areas indicate the arithmetic averages and standard deviations of the upper and lower limits of the additive area, respectively. Red dots indicate the experimental determination of the FIC_50_ pairs

Aiming to profiling the combinatorial potential of the selected Malaria Box set, the inhibitory effect of each compound’s association with artesunate was assessed. First, the combination profile of the control compounds was evaluated as follows: atovaquone in combination with proguanil, a pair of standard anti-malarial drugs with known synergistic effect [28], and artesunate with itself, which by the definition of Loewe additivity demonstrates an additive effect [29]. The isobolograms for both controls, atovaquone + proguanil (Fig. 4a) and artesunate + artesunate (Fig. 4b) indicate synergic and additive profiles, respectively. Based on that, the combination profile of the 11 selected compounds was assessed in combination with artesunate (Fig. 4c–m). Table 2 shows the fraction of experimental data inside, above, and below the additive zone for each investigated compound. The isobolograms for each pair of inhibitors were classified as additive (inside the additive zone), antagonistic (above the additive zone) or synergic (below the additive zone).

**Table 2:** Isobologram data and combination profiles of compounds **1**–**11** with artesunate. Numbers indicate how many FIC_50_ pairs were located in each isobologram region

Compound		Isobologram data			Combination profile
#	MMV code	Antagonistic	Additive	Synergic	
01	**MMV006172**	11 (92%)	1 (8%)	0	Antagonism
02	**MMV665971**	1 (8%)	12 (92%)	0	Additivity
03	**MMV007224**	8 (62%)	5 (38%)	0	Antagonism
04	**MMV666607**	12 (92%)	1 (8%)	0	Antagonism
05	**MMV020439**	11 (85%)	2 (15%)	0	Antagonism
06	**MMV665934**	11 (85%)	2 (15%)	0	Antagonism
07	**MMV007574**	7 (78%)	2 (22%)	0	Antagonism
08	**MMV085203**	8 (67%)	4 (33%)	0	Antagonism
09	**MMV085583**	0	13 (100%)	0	Additivity
10	**MMV018984**	8 (62%)	5 (38%)	0	Antagonism
11	**MMV019871**	12 (100%)	0	0	Antagonism
Artesunate		0	15 (100%)	0	Additivity

The combinatory profile evaluation of the selected compounds indicated that compounds **2** (Fig. 4d) and **9** (Fig. 4k) have an additive profile (Table 2), suggesting that the combination of these inhibitors with artesunate is favourable for the in vitro inhibitory activity. In light of that, the inhibitory activity against *P. falciparum*, cytotoxicity and selectivity of the pairs in an equimolar proportion were determined for both additive combinations (Table 1). The remaining compounds (**1**, **3**-**8**, **10**, and **11**) showed an antagonistic combinatory profile with artesunate (Fig. 4 and Table 2). Compounds with similar atomic connectivity and topological distance distribution, as verified by the clusterization process, consistently showed the same combination profile. For instance, the two most similar compounds within the dataset (**5** and **7**) showed the same combination profile with artesunate (antagonistic).

**Fig. 5:**
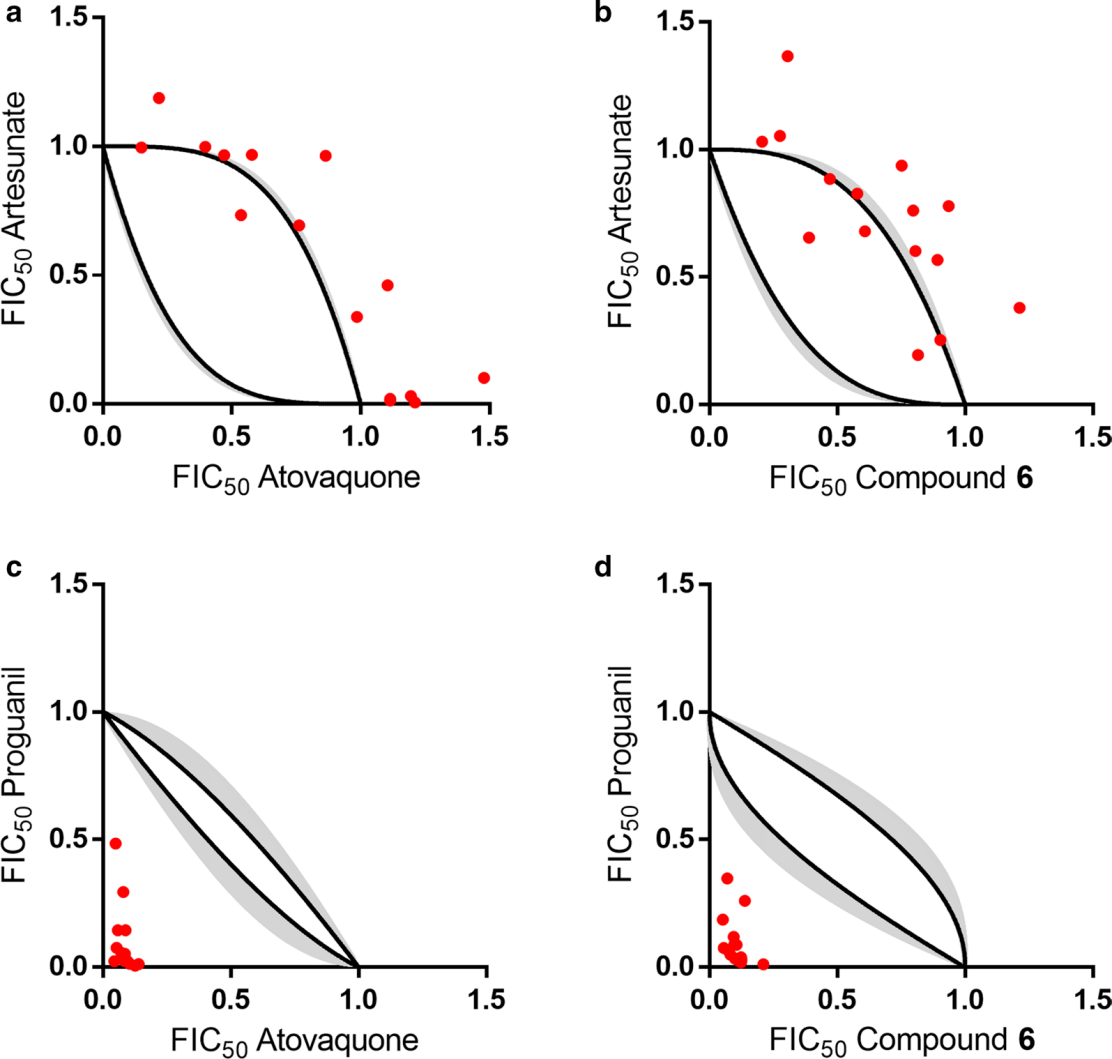
Isobolograms of **a** artesunate + atovaquone (antagonistic), **b** artesunate + **6** as a surrogate for atovaquone (antagonistic), **c** proguanil + atovaquone (synergic), and (**d**) proguanil + **6** as a surrogate for atovaquone (synergic)

Compound **6**, an indeno[1,2-c]pyridazin-5-one derivative, has been identified as an inhibitor of the mitochondrial electron transport chain targeting the bc_1_ complex [30]. This inhibitory property motivated the investigation of combination profile of **6** as a replacement for atovaquone (a known bc_1_ complex inhibitor) in drug association [31]. Thus, the isobolograms of **6 **+ artesunate and **6** + proguanil was compared with the control isobolograms (atovaquone + artesunate and atovaquone + proguanil) (Fig. 5). The combination of **6** with artesunate is antagonistic, as observed by the association of the control atovaquone + artesunate (Fig. 5a and b). On the other hand, the isobologram of **6** with proguanil indicated a highly synergic combination profile, which is in good agreement with the profile observed for the control combination of atovaquone and proguanil (Fig. 5c and d), a recommended drug combination therapy for malaria.

### In vivo evaluation of selected Malaria Box compounds

**Fig. 6:**
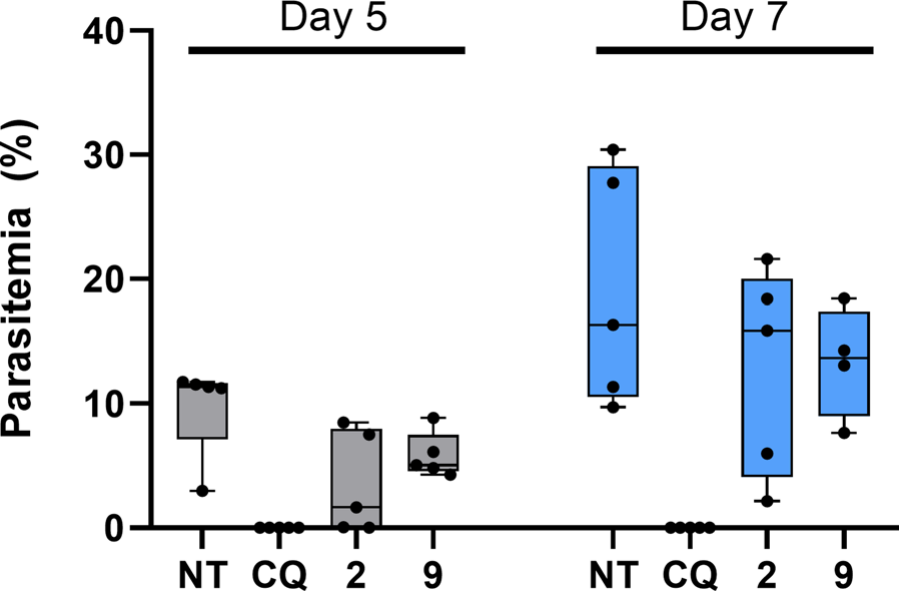
Percentage parasitemia on days 5 and 7 after infection. Compounds **2** and **9** were administered at 50 mg/kg by oral gavage. Chloroquine (CQ) was used as a positive control at 20 mg/kg, and data from untreated mice (NT) is presented for comparison

Motivated by the additive combination profile of compounds **2** and **9** with artesunate, their in vivo activity in *P. berghei*-infected mice was tested. Two groups of five infected mice were treated with 50 mg/kg of compounds **2** and **9**, respectively, via oral gavage for 3 consecutive days after infection. Parasitemia was evaluated on days 5 and 7 post-infection. The anti-malarial chloroquine was used as a positive control (20 mg/kg). Both compounds showed moderate in vivo activity, with compound **2** reducing parasitemia by 64% on day 5 and 33% on day 7 post-infection, whereas compound **9** reduced parasitemia by 40% on day 5 and 30% on day 7 post-infection (Fig. 6) (Table 3).

**Table 3:** Percentage of parasitaemia reduction after malaria treatment with **2** and **9**

Compound	Dose (mg/kg)	% of reduction, days after infection	
		5	7
**2**	50	64	33
**9**	50	40	30
Chloroquine	20	100	100

## Discussion

Based on the efficacy of artemisinin-based combination therapy, a new anti-malarial drug will preferentially be administered in combination with artemisinin derivatives or some of the other drugs used against malaria [32]. The combination of anti-malarial drugs not only delays the emergence and spread of drug resistance but also has the potential to interrupt the transmission of *P. falciparum* [32]. Moreover, this strategy simultaneously decreases the necessary amount of each component and the chances of a toxic effect during therapy [33]. Therefore, the identification of suitable partners for combination therapy is crucial to reduce the potential of drug resistance.

The Malaria Box compounds have not yet been assessed for potential interactions with artemisinin derivatives, a gold-standard drug class in human malaria treatment [30]. In this work, a chemoinformatic approach was used to select a small but representative dataset of 11 compounds from the Malaria Box to investigate the antiplasmodial properties and identify favourable combination partners with artesunate. The similarity indices between the selected compounds and artesunate were as low as 3%, indicating very dissimilar structural scaffolds. Next, the reported inhibitory activity of the selected set of inhibitors was confirmed and showed that the representative compounds have very low cytotoxicity against HepG2 cells (IC_50_^HepG2^ ranging from 3.21 to > 100 µM) and a high selectivity index (SI ranging from 15.1 to > 8300). Then, both the speed of action and the association profile with artesunate was investigated.

Compounds **1**–**3**, **5**, **7**, **9**–**11** showed fast inhibitory activity against in vitro parasite growth, whereas compounds **4**, **6**, and **8** showed slow inhibitory activity (Fig. 2). The set of selected compounds has a combination of favourable properties that contribute to high permeability through the membranes (Fig. 3). This finding suggests that the compounds’ permeation properties are not related to the speed of action, indicating that the observed differences in the speed of action might be due to the inhibitors’ mode of action [34]. In this context, the observed differences in the speed of action of artesunate, a fast anti-malarial agent, and compounds **4** (**MMV666607**), **6** (**MMV665934**) and **8** (**MMV085203**), which had slow antiplasmodial activity, suggest different mechanisms of inhibition against *P. falciparum*. Indeed, the slow inhibitory activity of compound **6** can be related to its molecular target (the bc_1_ complex) [30] because atovaquone, a bc_1_ complex inhibitor, is another slow anti-malarial agent [35]. The bc_1_ complex is a validated molecular target for new anti-malarial discovery and development [31, 36]. The combination profile of **6** as a surrogate molecule for atovaquone in drug combination was investigated (Fig. 5). The collected data indicated that **6** in combination with proguanil had a similar association profile to atovaquone combined with proguanil, possibly due to **6** and atovaquone sharing the same molecular target.

Due to lower resistance development rates, different molecular targets are the primary selection factor for combination therapy [37]. It is important to emphasize that a different mode of action is only one of the selection criteria. Here, the combinatory profile evaluation of the selected compounds with artesunate indicated two different readouts: antagonistic and additive (Fig. 4). Compounds **1**, **3**–**8**, **10** and **11** showed antagonistic combinatory profiles with artesunate. Specifically, compounds **4** (**MMV666607**, a hydrazineyl-1*H*-benzo[*d*]imidazole derivative), **6** (**MMV665934**, an indeno[1,2-c]pyridazin-5-one derivative) and **8** (**MMV085203**, a 3-(piperidin-1-yl)naphthalen-1(2*H*)-one derivative) are slow-acting *P. falciparum* inhibitors, thereby suggesting a different mode of action related to artesunate (fast-acting inhibitor). However, compounds **4**, **6**, and **8** showed antagonistic combinatory profiles with artesunate. Hence, the findings underscore the need for a more detailed investigation in addition to the assessment of the mode of action for the identification of favourable combination partners. On the other hand, the isobologram analysis of two of the fast-acting inhibitors, **2** (**MMV665971**, a 2,3-dihydro-5H-thiazolo[3,2-a]pyrimidine-6-carboxylate derivative) and **9** (**MMV085583**, a 1-hydroxy-dibenzo[b,e] [1, 4] diazepine derivative), indicated an additive profile in combination with artesunate, suggesting that the association of these compounds with artesunate is favourable for the in vitro inhibitory growth of *P. falciparum*.

To better characterize the antimalarial potential of compounds **2** and **9**, both inhibitors were selected for in vivo activity evaluation. Compounds **2** and **9** showed oral efficacy at 50 mg/kg in a mouse model of *P. berghei* malaria (64% and 40% reduction in parasitaemia on day 5 post-infection, respectively). The modest in vivo activity might be related to pharmacokinetics properties of the inhibitors. Previous pharmacokinetics studies revealed that compound **2** has poor oral bioavailability and high plasma protein binding (> 99%). Compound **9** showed a similar profile, but higher blood maximum concentrations [30]. Therefore, the improvement of the drug-like properties of **2** and **9** will enable the discovery of new promising ACT partners.

## Conclusions

In this work, a similarity analysis to select a set of 11 representative compounds from the Malaria Box was carried out. Next, the reported inhibitory activity of the Malaria Box compounds was confirmed and the selected compounds were shown to have very low cytotoxicity against HepG2 cells and a high selectivity index. Then, both the speed of action and the association profile of artesunate with the 11 selected compounds were investigated. Compounds **1**-**3**, **5**, **7**, **9**-**11** showed fast inhibitory activity of in vitro parasite growth, whereas compounds **4**, **6** and **8** were slow-acting in vitro inhibitors, in contrast to artesunate, a fast in vitro inhibitor [34]. Compound **6** (**MMV665934**), a bc_1_ complex inhibitor, exhibited antagonistic and synergic combination profiles when used in association with artesunate and proguanil, respectively. These findings are in good agreement with the combination profiles of atovaquone with the gold-standard anti-malarial drugs and could be explained by **6** and atovaquone sharing the same molecular target. Compounds **2** (**MMV665971**) and **9** (**MMV085583**) did not interfere with the high potency of artesunate and positively contributed to the inhibitory activities in the combination assays, thereby indicating favourable combination potential with artesunate. Both compounds showed promising in vivo activity potential. The findings shed light upon the relationships between the speed of action, molecular targets and combinatory profiles, and identified new hits as candidates for anti-malarial combination therapy.

## Supplementary information

**Additional file 1: Fig. S1.** Fragments of the dendrogram originated from the hierarchical clustering of 400 compounds from the Malaria Box, artesunate and atovaquone. Clusters 2, 16, 20, 23 and 25 are represented with the 2D structures from which they originated. **Fig. S2.** Fragments of the dendrogram originated from the hierarchical clustering of 400 compounds from Malaria Box, artesunate and atovaquone. Clusters 26, 27, 29, 32, 35 and 36 are represented with the 2D structures from which they originated. **Fig. S3.** Concentration-response curves of the selected compounds from the Malaria Box against *P. falciparum* (3D7). Curves in black and red refer to, respectively, the first and second experiment performed for the evaluation of the IC_50_ values. **Fig. S4.** Evaluated versus reported pIC_50_ values

## Data Availability

The datasets analysed during the current study are available from the corresponding author on reasonable request.

## Abbreviations

ACT: artemisinin-based combination therapy
IC_50_: half maximal inhibitory concentration
FIC_50_: fractional half maximal inhibitory concentration
MMV: Medicines for Malaria Venture
MTT: 3-(4,5-dimethylthiazol-2-yl)-2,5-diphenyltetrazolium bromide
PSA: polar surface area
SI: selectivity index
