# Supplementary material for: Antiplasmodial profile of selected compounds from Malaria Box: in vitro evaluation, speed of action and drug combination studies
Source: Malar J. 2019 Dec 30;18:447. doi: 10.1186/s12936-019-3069-3 (PMC6938011; doi:10.1186/s12936-019-3069-3)
Supplement: Supplementary file 1 — Additional file 1: Fig. S1. Fragments of the dendrogram originated from the hierarchical clustering of 400 compounds from the Malaria Box, artesunate and atovaquone. Clusters 2, 16, 20, 23 and 25 are represented with the 2D structures from which they originated. Fig. S2. Fragments of the dendrogram originated from the hierarchical clustering of 400 compounds from Malaria Box, artesunate and atovaquone. Clusters 26, 27, 29, 32, 35 and 36 are represented with the 2D structures from which they originated. Fig. S3. Concentration-response curves of the selected compounds from the Malaria Box against P. falciparum (3D7). Curves in black and red refer to, respectively, the first and second experiment performed for the evaluation of the IC50 values. Fig. S4. Evaluated versus reported pIC50 values [file 12936_2019_3069_MOESM1_ESM.docx]

**Additional file**

**
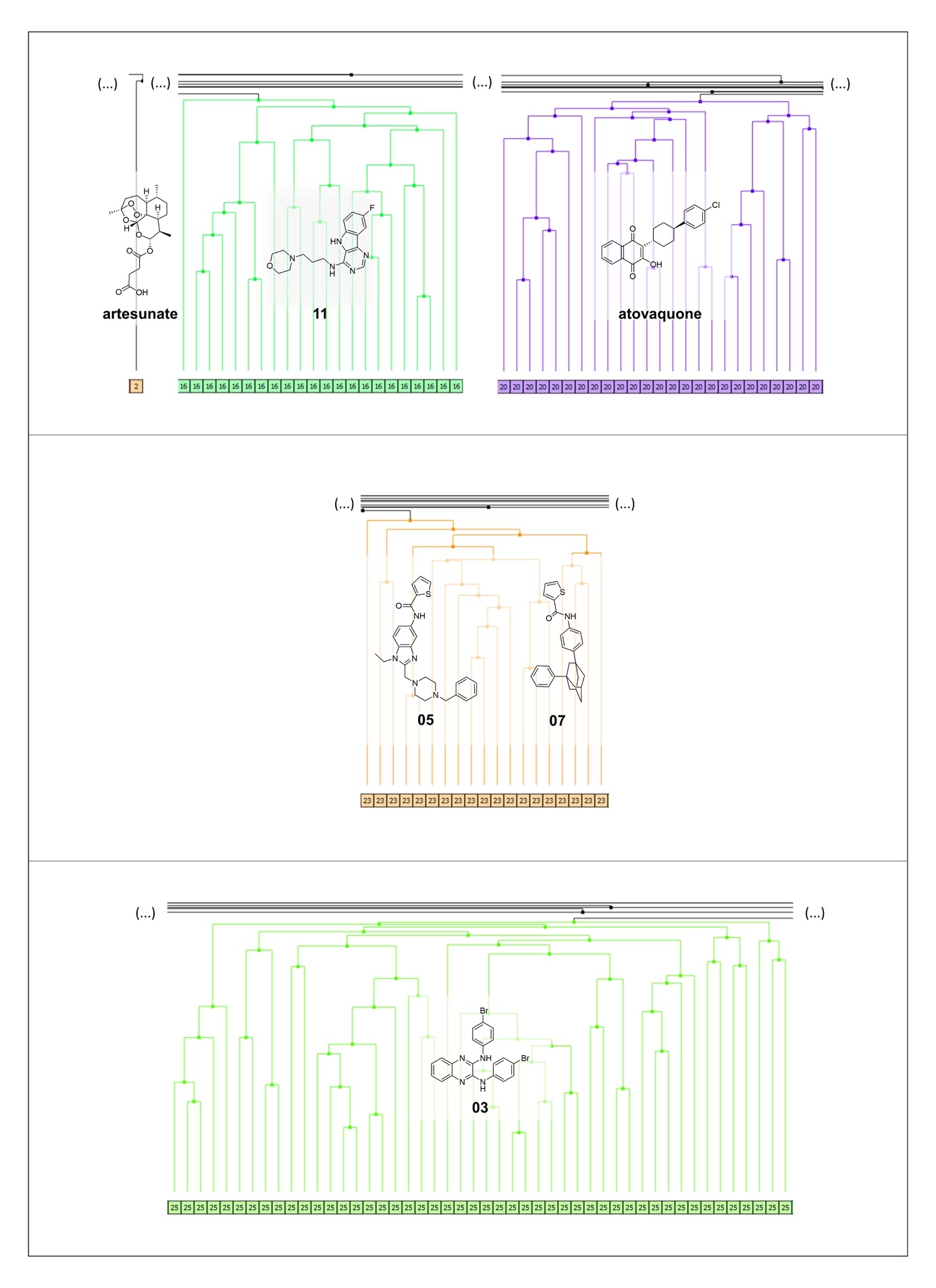
**

**Figure S1.** Fragments of the dendrogram originated from the hierarchical clustering of 400 compounds from the Malaria Box, artesunate and atovaquone. Clusters 2, 16, 20, 23 and 25 are represented with the 2D structures from which they originated.


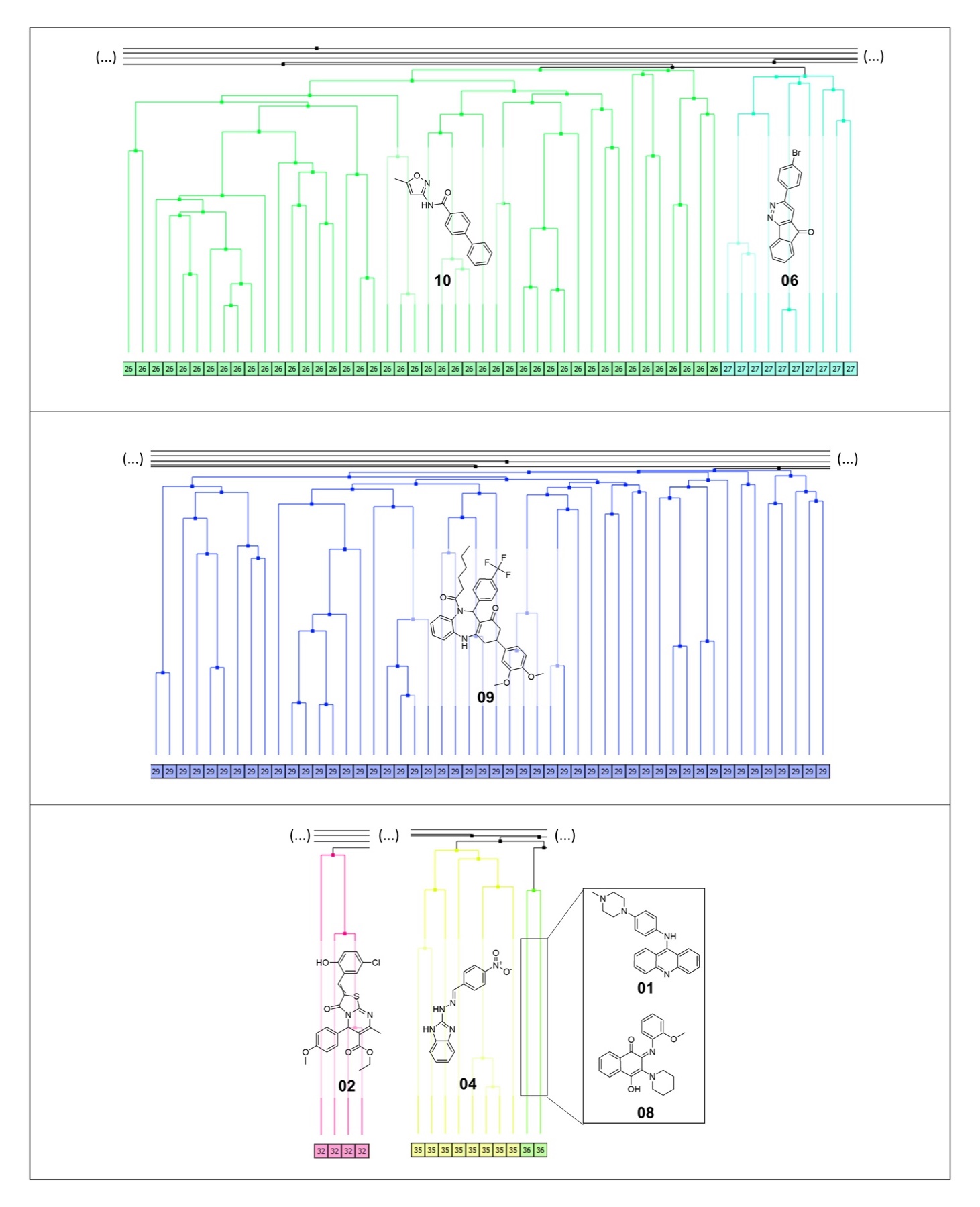


**Figure S2.** Fragments of the dendrogram originated from the hierarchical clustering of 400 compounds from Malaria Box, artesunate and atovaquone. Clusters 26, 27, 29, 32, 35 and 36 are represented with the 2D structures from which they originated.

**
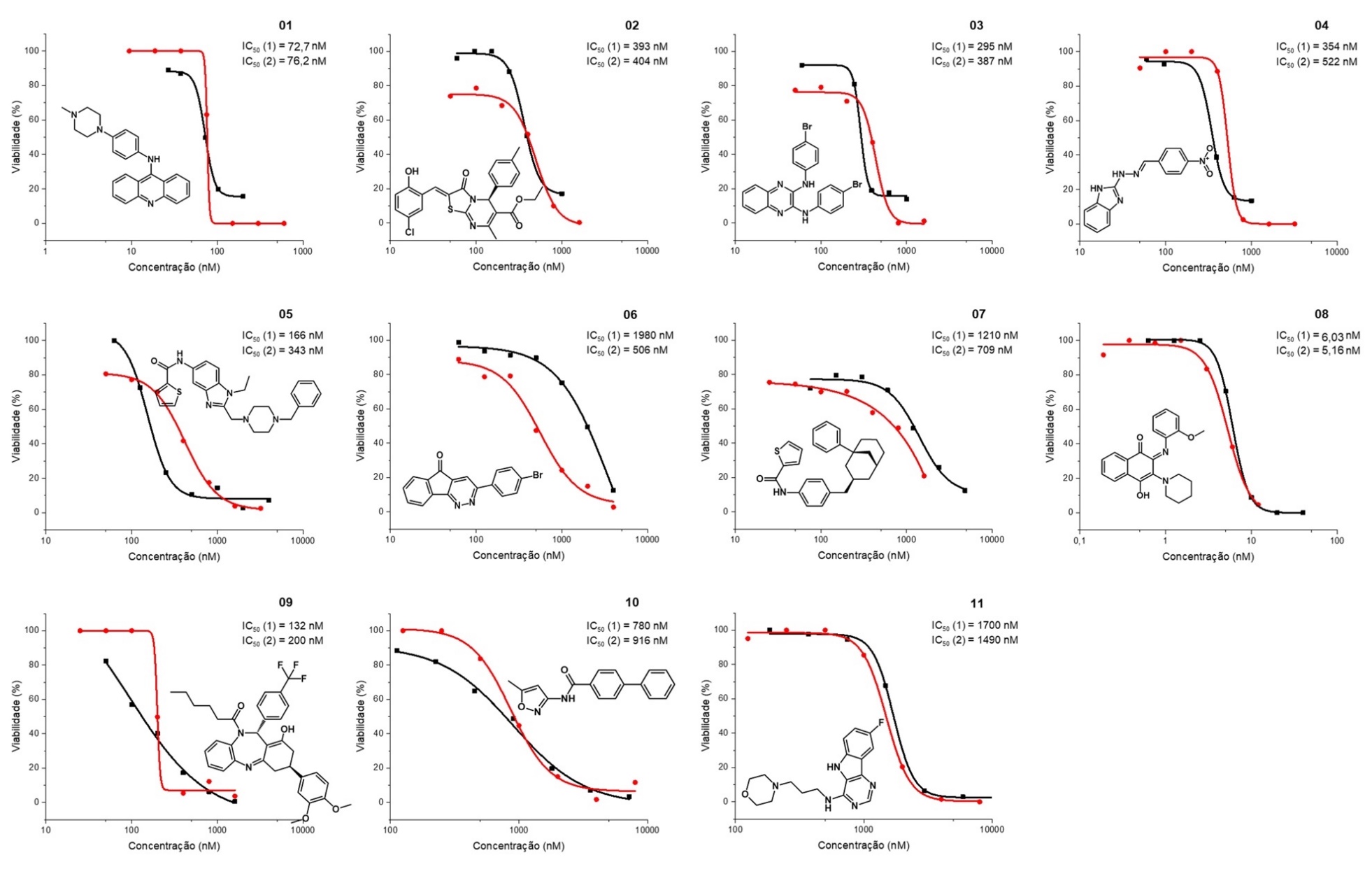
**

**Figure S3.** Concentration-response curves of the selected compounds from the Malaria Box against *P. falciparum* (3D7). Curves in black and red refer to, respectively, the first and second experiment performed for the evaluation of the IC_50_ values.


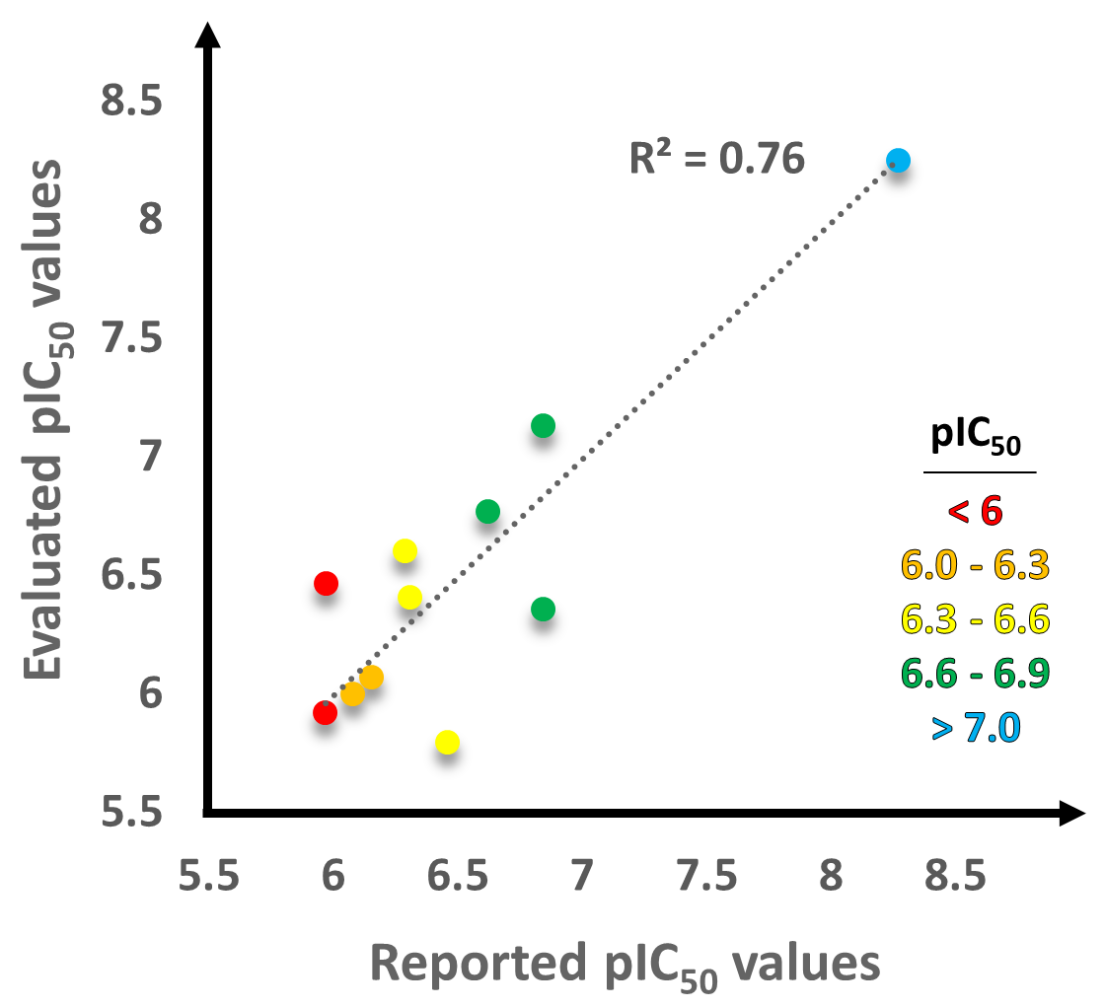


**Figure S4.** Evaluated versus reported pIC_50_ values
